# Nucleic Acid Content in Crustacean Zooplankton: Bridging Metabolic and Stoichiometric Predictions

**DOI:** 10.1371/journal.pone.0086493

**Published:** 2014-01-21

**Authors:** Francisco José Bullejos, Presentación Carrillo, Elena Gorokhova, Juan Manuel Medina-Sánchez, Manuel Villar-Argaiz

**Affiliations:** 1 Department of Ecology, Faculty of Sciences, University of Granada, Granada, Spain; 2 Water Research Institute, University of Granada, Granada, Spain; 3 Department of Applied Environmental Science, Stockholm University, Stockholm, Sweden; Estacion Experimental de Zonas Áridas (CSIC), Spain

## Abstract

Metabolic and stoichiometric theories of ecology have provided broad complementary principles to understand ecosystem processes across different levels of biological organization. We tested several of their cornerstone hypotheses by measuring the nucleic acid (NA) and phosphorus (P) content of crustacean zooplankton species in 22 high mountain lakes (Sierra Nevada and the Pyrenees mountains, Spain). The P-allocation hypothesis (PAH) proposes that the genome size is smaller in cladocerans than in copepods as a result of selection for fast growth towards P-allocation from DNA to RNA under P limitation. Consistent with the PAH, the RNA:DNA ratio was >8-fold higher in cladocerans than in copepods, although ‘fast-growth’ cladocerans did not always exhibit higher RNA and lower DNA contents in comparison to ‘slow-growth’ copepods. We also showed strong associations among growth rate, RNA, and total P content supporting the growth rate hypothesis, which predicts that fast-growing organisms have high P content because of the preferential allocation to P-rich ribosomal RNA. In addition, we found that ontogenetic variability in NA content of the copepod *Mixodiaptomus laciniatus* (intra- and interstage variability) was comparable to the interspecific variability across other zooplankton species. Further, according to the metabolic theory of ecology, temperature should enhance growth rate and hence RNA demands. RNA content in zooplankton was correlated with temperature, but the relationships were nutrient-dependent, with a positive correlation in nutrient-rich ecosystems and a negative one in those with scarce nutrients. Overall our results illustrate the mechanistic connections among organismal NA content, growth rate, nutrients and temperature, contributing to the conceptual unification of metabolic and stoichiometric theories.

## Introduction

The metabolic theory of ecology (MTE) and biological stoichiometry (BS) have greatly advanced our understanding of the factors that control ecological processes [1], [2]. While the MTE focuses on energy as the primary currency of metabolism [3], [4], BS studies the balance of energy and multiple chemical elements in living systems [1], [5].

Both theories place special emphasis on unraveling the mechanistic basis of individual metabolism and growth as it affects the energy flux, and the storage and turnover rates of elements in ecosystems [1], [2]. The growth rate (GR), defined as an increase in size (biomass) or protein content per unit of time, is one of the most relevant ecological traits, because it is an integrating parameter of overall life history strategy [6]. It not only affects other important life-history traits and ecological features, such as the age at first reproduction or the ability to inhabit temporary habitats [7], but also serves as a measure of animal fitness, because organisms must grow to reproduce [5], [6]. The growth rate hypothesis (GRH), a central concept of BS, proposes that organisms lacking major phosphorus (P) storage capacity have elevated demands for increased P allocation to P-rich ribosomal RNA under rapid growth. This drives variation in the P content (and therefore C:P and N:P ratios) in these organisms and establishes the close connection among individual growth, ribosomal metabolism, and elemental composition [5]. It also provides the rationale for the use of RNA-based biomarkers, e.g., RNA content (as % of dry weight [%RNA]) or RNA:DNA ratio, as proxies for GR in various species, including zooplankton (e.g. [8], [9]).

It has been suggested that natural selection operating on GRs drives differences in the body P content of copepods and cladocerans [5], [10], [11]. Fast-growing cladocerans possess a high P content due to the high demand for P allocation to RNA for ribosome and protein biosynthesis [12], [13]. In contrast, a more relaxed selection for fast growth has been associated with lower P content in copepods [10], [11], [14].

Considerable interest in the genetic basis of GRH has emerged since the development of genomics earlier this century. A genetic approach offers an opportunity to study the way natural selection simultaneously operates on genome size and GR, which are traditionally studied separately in evolutionary biology [5]. Various studies have demonstrated that increased GR and the associated increase in transcriptional capacity for ribosomal RNA production are positively associated with the length and content of ribosomal DNA intergenic spacers and/or copy number [15], [16], [17]. However, this pattern appears contrary to the pervasive association of high GR, RNA, and P contents with small genome size in rapid-growth organisms such as cladocerans, particularly *Daphnia* species. To explain this paradox, the P-allocation hypothesis (PAH) proposes that small genomes in cladocerans are the consequence of P allocation from DNA (mainly non-coding DNA) to RNA under sustained selection for rapid growth in P-limited environments [13], [18]. Conversely, copepods have lower P content [10] and larger genomes than cladocerans, resulting in up to 15-fold lower RNA:DNA ratios [13]. Accordingly, a strong selection pressure to reallocate P from DNA to RNA would not be expected in low-P demanding copepods. However, this proposition is challenged by reports that larval stages of copepods (nauplii) have high demands for P to sustain high GRs [14], [19], [20]. Although the PAH has been suggested as a plausible mechanism for the evolution of reduced genome size in eukaryotes [13], [18], the generality of these arguments awaits empirical evidence; further corroboration is required in a broader context under varied ecological and physiological conditions, including the study of intraspecific differences associated with ontogenetic development and the role of environmental constraints such as temperature in determining nucleic acid (NA) content. According to the MTE, the metabolic rate of an animal varies with body size and temperature [2], which should in turn influence the GR [3]. Thus, specific metabolic rates would tend to be higher in organisms operating at warm temperatures than in organisms of similar size operating at colder temperatures [3]. However, the MTE has been criticized on theoretical grounds, because it does not address how the availability of nutrients may account for much of the variation in temperature dependence processes [2], [4]. Thus, if MTE and stoichiometric predictions were valid, the positive relationship between NA composition and temperature, predicted by the MTE, would be expected to decline with increasing nutrient limitation.

We tested the PAH by determining the P and NA content and RNA:DNA ratio of several crustacean zooplankton species in 22 high mountain lakes (Sierra Nevada and the Pyrenees mountains, Spain). The GRH was tested by examining the relationship between RNA and P content in the studied zooplankton taxa and among RNA, P, and GR across the life cycle of the copepod *Mixodiaptomus laciniatus*. Finally, we studied the NA content in all zooplankton species in order to test the MTE and BS predictions for the combined effect of temperature and nutrients on the GR. Extreme low-nutrient alpine environments are ideal model systems for testing these hypotheses. First, their oligotrophic to ultra-oligotrophic status implies a strong food limitation for zooplankton, which may play a major ecological role in determining species composition [21], [22]. Second, seston food for zooplankton is generally low in P relative to C in clear oligotrophic lakes [23], an essential requisite for testing the PAH and GRH. Finally, short ice-free periods, high levels of ultraviolet radiation (UVR) [24], low temperatures, and fluctuating hydrological regimes [25] constitute strong selective pressures for a high GR [26], [27].

## Materials and Methods

The study was carried out in 22 high mountain lakes (1600–3100 m above sea level) in the National Park of Sierra Nevada and the Pyrenees (Spain). These lakes are small, shallow, and highly transparent, with absent or very scarce littoral vegetation (see Table S1 in Supporting Information). Research permits for this study were provided by the National Parks of Sierra Nevada and Aigüestortes i Estany Sant Maurici (Spain).

### Field Sampling

In the summer of 2005, physical, chemical, and biological data for each lake were collected between 6 July and 25 August (Table S1). In addition, lake Laguna de la Caldera was sampled at 3- to 6-week intervals during the ice-free periods (June-October) in 2005, 2006, and 2007.

Temperature and light (UVR at 305, 320, and 380 nm [UVR_305, 320, and 380 nm_] and photosynthetic active radiation [PAR]) profiles were measured along the water column using a Biospherical Instrument Compact 4-Channel Radiometer (Biospherical Instruments Inc., San Diego, California, USA). Each lake was characterized by the mean temperature of the water column and the diffuse attenuation coefficient (*K_d_*) at each wavelength, calculated from the slope of the linear regression of the natural logarithm of downwelling irradiance vs. depth. An integrated extinction coefficient of UVR (*K_d UVR_*) was calculated as the mean extinction coefficient of the three UVR wavelengths (305, 320, and 380 nm) (Table S1).

Chemical and biological samples were taken with a 6-L Van Dorn sampler at the deepest point of the lake. When possible, water from up to four depths (0.5 m below surface, 0.5 m above the bottom, and two intermediate sampling depths) was mixed in a 5-L bucket, and subsamples were taken in triplicate for total P (TP). After removing zooplankton by sieving water through a 40-µm mesh, another set of subsamples was taken in triplicate for P (hereafter, TP′), chlorophyll *a* (Chl *a*), seston carbon (C), nitrogen (N), P, and primary production measurements.

Zooplankton samples for abundance and biomass determinations were obtained after sieving 24 L of water from the sampling depths through a 40-µm mesh and were preserved in 4% formaldehyde. Zooplankton was identified and counted with the aid of an inverted microscope at 100× magnification. For each sample, the length of up to 20 individuals of each cladoceran species or copepod developmental stage was measured with an image analysis system (Quantimet 500, Leica, Wetzlar, Germany). The crustacean zooplankton biomass was estimated by using length-weight regressions specifically developed for the copepods *Acanthocyclops vernalis* (copepodites by Rosen [28]; adults by Bottrell et al. [29]); *Cyclops abyssorum* (nauplii by Rosen [28]; copepodites and adults by Ventura [30]); *Diaptomus cyaneus* (nauplii by Rosen [28]; copepodites and adults by Ventura [30]); *Eudiaptomus vulgaris* (nauplii by Rosen [28]; copepodites and adults by Persson & Ekbolm [31]); *Mixodiaptomus laciniatus* (nauplii, copepodites, and adults by Carrillo et al. [14]); and the cladocerans *Alona affinis* [32], *Chydorus sphaericus* [28], *Daphnia longispina* [29], and *Daphnia pulicaria* [30]. For rotifers and ciliates, we considered the individual weights published by Bottrell et al. [29], Dumont et al. [32], and Walz [33].

Additional samples of zooplankton for P and NA determinations were collected by vertical hauls of a 40-µm mesh net and transported chilled in lake water to the laboratory. In the laboratory, zooplankton was concentrated by sieving through a 40-µm mesh and diluted to 1 L with 0.7-µm filtered lake water. For the analysis of P content, the species of live individuals was identified with the aid of an inverted microscope and sorted into precombusted (1 h at 550°C) 1.0-µm glass fiber filters (Whatman GF/B). When possible, samples containing 30–50 individuals of *Cyclops abyssorum*, 10–20 of *Diaptomus cyaneus*, 5–15 of *Alona affinis*, 20–25 of *Daphnia longispina*, and 5–15 of *Daphnia pulicaria* were isolated. We distinguished among ontogenetic stages and between adult genders in copepods and between non-ovigerous and ovigerous reproductive statuses in copepods and cladocerans. Three replicates per lake were collected for the most abundant species or copepod stages, whereas single or duplicate samples were collected when these were less abundant. Simultaneously, samples were taken and fixed in 4% formaldehyde for later individual body size measurements and, after biomass conversions, for estimation of total P content as % of dry weight (%P). GR and %P data for *Mixodiaptomus laciniatus* were obtained from Carrillo et al. [14] and the developmental time (DT) for each ontogenetic stage from Cruz-Pizarro [34]. For NA analysis, up to 20 individuals from each species were sorted into 1.5 mL Eppendorf tubes containing 300 µL RNA*later* (Ambion Inc., Austin, Texas, USA), and stored at −80°C until analysis [35]. The number of collected individuals was 10–102 for each species and lake combination, and 10–56 for each *Mixodiaptomus laciniatus* ontogenetic stage and sampling day.

Primary production was measured with the ^14^C method proposed by Steeman-Nielsen [36]. Sets of four 50-mL quartz flasks (three clear and one dark) containing the water with 0.37 MBq NaH^14^CO_3_ (specific activity: 310.8 MBq mmol^−1^; NEN DuPont, Boston, Massachusetts, USA) were incubated *in situ* for 4 h symmetrically distributed around noon at a depth where the UVR was 75% the surface value. All flasks were held horizontally during the incubations. Primary production was measured as total organic carbon (TOC) by acidifying a 4-mL subsample in a 20-mL scintillation vial with 100 µL of 1 N HCl and allowing the vial to stand open in a hood for 24 h (no bubbling), as recommended by Lignell [37]. Particulate primary production >1.0 µm (particulate organic carbon >1.0 µm, POC_1_) was determined by filtering an aliquot of 40 mL through 1.0 µm pore-size Nucleopore filters of 25-mm diameter, applying a low pressure (<100 mm of Hg) to minimize cell breakage. Filters were placed in scintillation vials and the dissolved inorganic ^14^C was removed by adding 100 µL of 1 N HCl. We added 16 mL of liquid scintillation cocktail (Ecoscint A; National Diagnostics Inc, Charlotte, North Carolina, USA) to the vials, and the radioactivity was counted at 12 h in a Beckman LS 6000 TA scintillation counter equipped with autocalibration (Beckman Instruments Inc., Fullerton, California, USA). The total CO_2_ in the lake water was calculated from the alkalinity and pH measurements [38]. In all calculations, dark values were subtracted from corresponding light values.

### Chemical and Biological Analyses

TP and TP′ were determined by analyzing 50-mL aliquots with the acid molybdate technique after digestion with a mixture of potassium persulfate, boric acid, and sodium hydroxide at 120°C for 30 min [38]. Up to 300 mL (for seston C and N) or 400 mL (for seston P) per replicate were filtered through precombusted (1 h at 550°C) 1.0-µm glass fiber filters (Whatman GF/B) at low pressure (<100 mm Hg). Filters containing seston C and N were dried (24 h at 60°C), and kept desiccated until C and N analysis using a Perkin-Elmer model 2400 CHN elemental analyzer (Perkin-Elmer Corporation, Waltham, Massachussets, USA). Seston and zooplankton P were analyzed following the method described for TP and TP′. Blanks and standards were performed in all procedures. Seston C:N:P ratios were calculated on a molar basis. Chl *a* was measured fluorimetrically after filtration of 300 mL per replicate through 0.7-µm glass fiber filters (Whatman GF/F) at low pressure (<100 mm Hg) and 24-h pigment extraction in 90% acetone in the dark at 4°C. A Chl *a* standard (Chl *a* from algae; Fluka/Sigma-Aldrich, Buchs, Switzerland) was used to transform the fluorescence data into Chl *a* concentrations.

NA analysis was carried out with a microplate fluorometric high-range assay with RiboGreen using length-measured individual zooplankters after extraction with N-laurylsarcosine followed by RNase digestion [39]. This method allowed for individual zooplankton NA measurements. The following working reagents were used: RiboGreen™ RNA Quantitation Kit (Invitrogen Corporation, Carlsbard, California, USA); RNase DNasefree (working solution 5 µg mL^−1^; Q-biogen, Weston, Massachussets, USA); N-lauroysarcosine (Sigma-Aldrich, Saint Louis, Missouri, USA); Tris-EDTA buffer (Q-biogene). Fluorescence measurements were performed using a FLUOstar Optima fluorometer (microplate reader, filters: 485 nm for excitation and 520 nm for emission; BMG Labtechnologies, Ortenberg, Germany) and black solid flat-bottom microplates (Greiner Bio-One GmbH, Frickenhausen, Germany). The plate was scanned with a 0.2-s well measurement time, making 10 measurements per well, before and after RNase digestion (30 min under dark conditions at 37°C). Fluorescence measurements were converted into RNA and DNA concentrations by using standard curves for RNA (16S and 23S from *Escherichia coli*, component C of the RiboGreen Kit) and DNA (calf thymus; Sigma-Aldrich). RNA and DNA contents were expressed relative to dry weight (%RNA and %DNA) after biomass conversions using the above-mentioned length-weight regressions and also as RNA:DNA ratio. The %P allocated to RNA (%P-RNA) and DNA (%P-DNA) were calculated by multiplying the content of each NA by its P proportion (0.085 for RNA and 0.089 for DNA; [40]), while the % of P allocated to total NAs (%P-TNAs) was calculated as the sum of %P-RNA and %P-DNA. For *Mixodiaptomus laciniatus* from lake Laguna de la Caldera, we also calculated the percentage ratio between the P content allocated to NAs (%P-RNA, %P-DNA, and %P-TNAs) and the total P content of the organism (%P) as a measure of the relative P investment in NAs (hereafter, ‘relative P investment index’ for RNA [RPII_RNA_], DNA [RPII_DNA_] and TNAs [RPII_TNAs_]).

### Statistical Analyses

Intergroup, inter-, and intraspecific differences in body size, %RNA, %DNA, RNA:DNA ratio, %P-TNAs, and %P were analyzed by general linear models. Nested design analysis of variance (nested design ANOVA) was performed to test for the effects of group (copepods vs. cladocerans), species and lake of origin, and analysis of covariance (ANCOVA) for the effects of group, species, lake temperature, TP′, and temperature×TP′ interaction. We also used ANCOVA to test for the effects of (i) group, species, and lake temperature below- and above-median TP′, and (ii) group, species, and lake TP′ below- and above-median temperature on %RNA. For these analyses, group and species were considered as fixed effects factors with species nested within group, lake as a random factor and temperature and TP′ as covariates. One-way ANOVA was used to analyze intraspecific differences due to ontogeny and gender (male vs. female) for copepods and due to female reproductive status (non-ovigerous vs. ovigerous) for copepods and cladocerans. The effect of sampling year was also included as a random factor in the main effects ANOVA to explore intraspecific differences due to ontogeny, gender and female reproductive status in the copepod *Mixodiaptomus laciniatus* from lake Laguna de la Caldera. When significant effects were found, pairwise comparisons were made with Tukey’s HSD post-hoc tests. The low number of replicates for %P precluded the study of the effects of lake of origin, temperature, and TP′ for all species. Therefore, %P data collected from different lakes were pooled for analysis comparing groups and species. Given that all samples were collected in 1997 (see [14]), the effect of sampling year was not considered for testing %P in the *Mixodiaptomus laciniatus* study. We based the statistical analyses on reciprocal square root- and natural log-transformed variables for all species and for *Mixodiaptomus laciniatus*, respectively, to induce homogeneity of variances or at least reduce or eliminate the correlations between the means and the variances ([41]; see also STATISTICA electronic manual [42]).

Partial regression plots were used to display the relationships of (i) temperature with %RNA at below- and above-median TP′, and (ii) TP′ with %RNA at below- and above-median temperature in all species, following recommendations by Moya-Laraño & Corobado [43]. Simple linear regression analyses were used to test (i) the relationship between %RNA and %P in all species and stages of *Mixodiaptomus laciniatus* and between GR and %P and between GR and %RNA in stages of *Mixodiaptomus laciniatus*; (ii) the relationship between body size and DT; and (iii) the relationships of body size and DT (independent variables) with all RPIIs (dependent variables) in *Mixodiaptomus laciniatus*. A homogeneity of slopes model (ANCOVA) was used to test the effect of NA (categorical factor) across body sizes (continuous predictor variable) on RPII [44]. Assumptions of normality and homoscedasticity for the parametric analyses were checked (Kolmogorov-Smirnov and Shapiro-Wilk's W tests, Cochran’s and Levene’s tests, respectively). STATISTICA 10 for Windows software [42] was used for the statistical analyses.

## Results

Most of the lakes sampled in this survey were oligotrophic as indicated by the nutrient (TP, TP′), and algal standing stock variables (Chl *a*, seston C, TOC, POC_1_). Distributions of these variables were skewed. For instance, Chl *a* was in the range 0.25–11.85 µg L^−1^, with 82% of observations <5 µg L^−1^; or seston C was in the range of 126–1032 µg C L^−1^, with 86% of observations <500 µg C L^−1^). Food quality for consumers was high, with seston C:P and C:N ratios of 104–364 (<306 for 86% of lakes) and 6–12 (<9.6 for 95% of lakes), respectively (Table 1).

**Table 1:** Descriptive statistics for the trophic variables analyzed in our set of 22 high mountain lakes.

	Minimum	1^st^ Quartile	Median	3^rd^ Quartile	Maximum
TP (µg P L^−1^)	4.0	7.9	10.6	13.8	34.3
TP′ (µg P L^−1^)	4.0	7.4	10.2	13.5	34.2
Chl *a* (µg L^−1^)	0.2	0.7	1.3	4.6	11.8
Seston C (µg C L^−1^)	126.3	164.6	269.1	403.0	1032.2
Seston C:N ratio (molar)	5.8	7.1	7.5	8.5	11.7
Seston C:P ratio (molar)	104.3	164.1	235.8	299.8	363.7
TOC (µg C L^−1^ h^−1^)	0.3	1.8	3.7	9.4	30.4
POC_1_ (µg C L^−1^ h^−1^)	<0.1	0.3	1.4	3.2	16.7

Variables: TP, total phosphorus; TP′, TP<40 µm; Chl *a*, chlorophyll *a*; seston C; seston C:N ratio; seston C:P ratio; TOC, total organic carbon; POC_1_, particulate organic carbon >1.0 µm. Units are given in brackets.

Zooplankton biomass varied from <1 to a maximum of 686 µg dry weight L^−1^ (Fig. S1A). Mesozooplankton was dominant in most lakes and mainly comprised copepods in the lakes of Sierra Nevada, particularly *Mixodiaptomus laciniatus,* and cladocerans in the lakes of the Pyrenees (Fig. S1B). A substantial presence of microzooplankton was also observed in lakes Lagunillo Grande de la Virgen, Estany Llong, and Estany dels Barbs. *Diaptomus cyaneus* (Laguna de la Gabata), *Chydorus sphaericus* (Laguna Hondera), *Cyclops abyssorum*, *Alona affinis* and *Daphnia longispina* (Estany Baix de Montcasau and Estany de la Munyidera) were also present, although only sporadically and in extremely low abundance (Fig. S1B).

### Intergroup and Interspecific Variability in NAs and P

**Figure 1:**
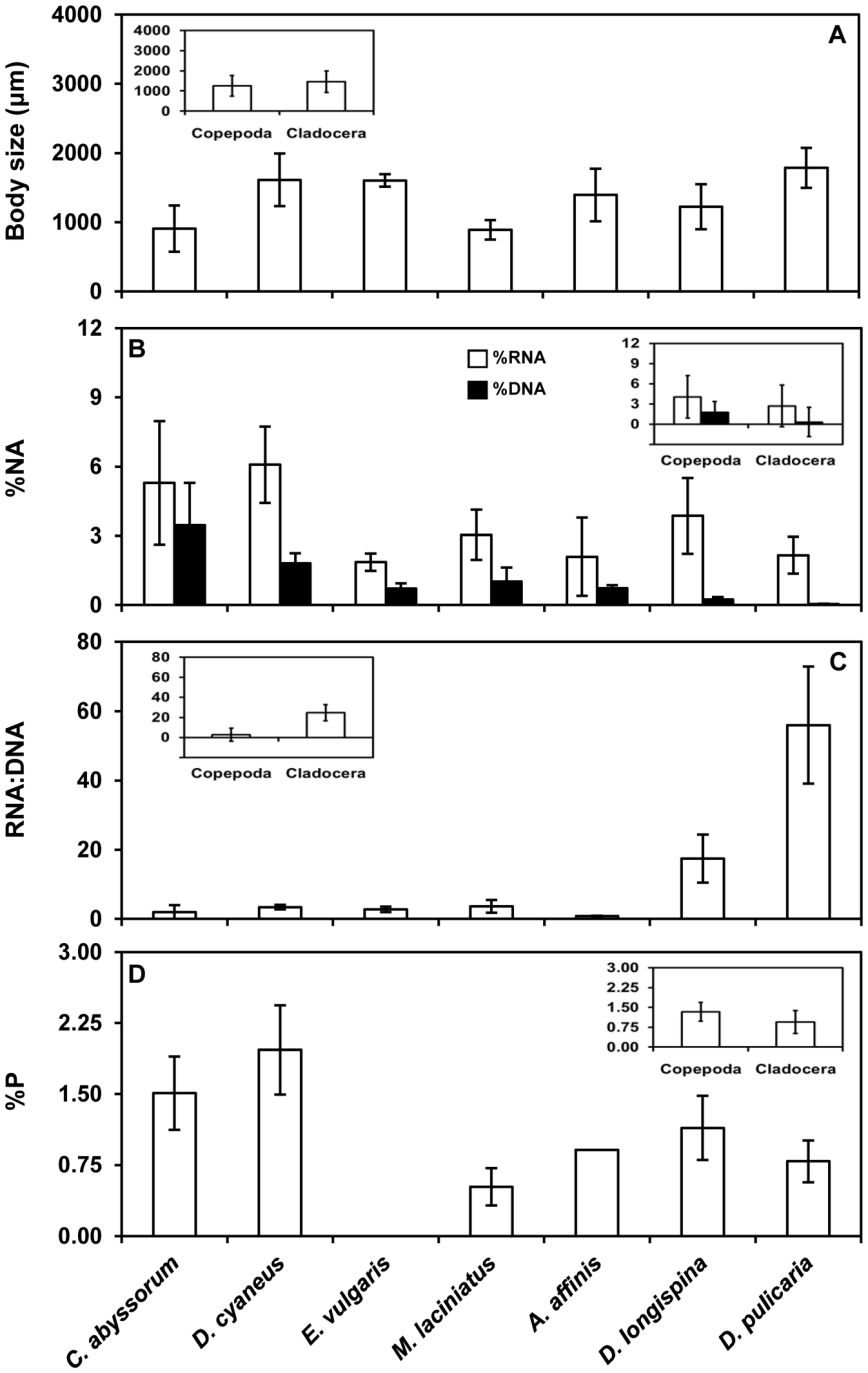
Body size, nucleic acid content, RNA:DNA ratio and total phosphorus content for investigated zooplankton species. (A) Body size, (B) nucleic acid (NA) content (% of dry weight, %NA), (C) RNA:DNA ratio, (D) total phosphorus (P) content (% of dry weight, %P) for each crustacean species of copepods [*Cyclops (C.) abyssorum*, *Diaptomus (D.) cyaneus*, *Eudiaptomus (E.) vulgaris*, *Mixodiaptomus (M.) laciniatus*] and cladocerans [*Alona (A.) affinis, Daphnia (D.) longispina*, *Daphnia (D.) pulicaria*]. Insets represent (A) body size, (B) %NA, (C) RNA:DNA ratio, and (D) %P for species grouped into copepods (copepoda) and cladocerans (cladocera). Columns are mean values, and error bars are standard deviations. Note that lack of %P data for *Eudiaptomus vulgaris* was due to the inability to collect a sufficient number of individuals for reliable estimations.

The intergroup and interspecific NA variability among seven crustacean zooplankton taxa was examined in a large number of samples (>400 individuals). While the %RNA and %DNA values were consistently higher in copepods than cladocerans (Tables 2, 3; see inset in Fig. 1B), the RNA:DNA ratio was >8-fold higher in the latter (Tables 2, 3; see inset in Fig. 1C). Among copepods, *Cyclops abyssorum* and *Diaptomus cyaneus* showed significantly elevated values of both NAs (Fig. 1B; Table 4). In contrast, *Daphnia* species showed strikingly low %DNA values (Fig. 1B; Table 4), which resulted in considerably higher RNA:DNA ratios relative to the other species (Fig. 1C; Table 4).

**Table 2:** Results of nested design ANOVA to analyze intergroup (copepoda vs. cladocera) and interspecific differences, and random effects of lake of origin in reciprocal square root-transformed variables: body size (µm), RNA and DNA contents (% of dry weight, %RNA and %DNA), RNA:DNA ratio, phosphorus (P) allocated to total nucleic acids (TNAs), and total P content (% of dry weight, %P-TNAs and %P).

Response variable	Source of variation	df	*F*	*p*-value
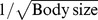	Group	**1**	**9.71**	**0.002**
	Species (Group)	**3**	**14.62**	**<0.001**
	Lake	**7**	**18.80**	**<0.001**
	Error	626		
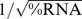	Group	**1**	**50.72**	**<0.001**
	Species (Group)	**3**	**63.11**	**<0.001**
	Lake	**7**	**29.82**	**<0.001**
	Error	390		
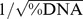	Group	**1**	**646.90**	**<0.001**
	Species (Group)	**3**	**181.87**	**<0.001**
	Lake	**7**	**3.530**	**0.001**
	Error	373		
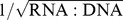	Group	**1**	**44.06**	**<0.001**
	Species (Group)	**3**	**73.83**	**<0.001**
	Lake	**7**	**11.03**	**<0.001**
	Error	321		
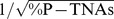	Group	**1**	**93.61**	**<0.001**
	Species (Group)	**3**	**49.79**	**<0.001**
	Lake	**7**	**15.19**	**<0.001**
	Error	321		
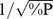	Group	1	0.22	.n.s.
	Species (Group)	**4**	**17.66**	**<0.001**
	Lake			
	Error	30		

Species (Group) denotes Species nested within Group. Reported are: degrees of freedom (df), *F*-test results (*F*), and significance level (*p*-value). Significant results (*p*-value <0.05) are indicated in bold; n.s., not significant.

**Table 3:** Results of ANCOVA to analyze intergroup (copepoda vs. cladocera) and interspecific differences, and the single and interactive effects of lake temperature and phosphorus (TP′) as covariates in reciprocal square root-transformed variables: body size (µm), RNA and DNA contents (% of dry weight, %RNA and %DNA), RNA:DNA ratio, phosphorus (P) allocated to total nucleic acids (TNAs), and total P content (% of dry weight, %P-TNAs and %P).

Response variable	Source of variation	df	*F*	*p*-value	*PV*
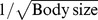	Group	**1**	**10.08**	**0.002**	**0.95**
	Species (Group)	**5**	**30.61**	**<0.001**	**14.46**
	Temperature	**1**	**64.62**	**<0.001**	**6.10**
	TP′	**1**	**79.31**	**<0.001**	**7.49**
	Temperature×TP′	**1**	**76.16**	**<0.001**	**7.19**
	Error	630			
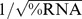	Group	**1**	**61.63**	**<0.001**	**7.51**
	Species (Group)	**5**	**56.32**	**<0.001**	**34.31**
	Temperature	**1**	**51.90**	**<0.001**	**6.32**
	TP′	**1**	**75.27**	**<0.001**	**9.17**
	Temperature×TP′	**1**	**67.25**	**<0.001**	**8.19**
	Error	394			
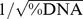	Group	**1**	**588.95**	**<0.001**	**18.03**
	Species (Group)	**5**	**177.81**	**<0.001**	**27.21**
	Temperature	1	2.54	.n.s.	0.08
	TP′	**1**	**4.62**	**0.032**	**0.14**
	Temperature×TP′	**1**	**4.07**	**0.044**	**0.12**
	Error	377			
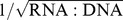	Group	**1**	**17.47**	**<0.001**	**1.05**
	Species (Group)	**5**	**66.51**	**<0.001**	**20.06**
	Temperature	**1**	**24.86**	**<0.001**	**1.50**
	TP′	**1**	**22.28**	**<0.001**	**1.34**
	Temperature×TP′	**1**	**21.24**	**<0.001**	**1.28**
	Error	325			
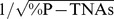	Group	**1**	**120.48**	**<0.001**	**13.90**
	Species (Group)	**5**	**62.02**	**<0.001**	**35.77**
	Temperature	**1**	**15.11**	**<0.001**	**1.74**
	TP′	**1**	**28.41**	**<0.001**	**3.28**
	Temperature×TP′	**1**	**24.44**	**<0.001**	**2.82**
	Error	325			
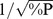	Group	1	0.22	n.s.	00.22
	Species (Group)	**4**	**17.66**	**<0.001**	**70.04**
	Temperature				
	TP′				
	Temperature×TP′				
	Error	30			

Species (Group) denotes Species nested within Group. Reported are: degrees of freedom (df), *F*-test results (*F*), significance level (*p*-value), and percentage of variance (*PV*) calculated as (sum of squares of treatment/total sum of squares)×100. Significant results (*p*-value <0.05) are indicated in bold; n.s., not significant.

**Table 4:** Results of Tukey’s HSD post-hoc tests to analyze interspecific differences in reciprocal square root-transformed variables: body size (µm), RNA and DNA contents (% of dry weight, %RNA and %DNA), RNA:DNA ratio, phosphorus (P) allocated to total nucleic acids (TNAs), and total P content (% of dry weight, %P-TNAs and %P).

Species	Species	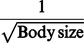	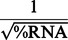	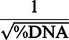	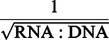	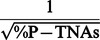	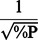
		*p*-value	*p*-value	*p*-value	*p*-value	*p*-value	*p*-value
*Cyclops abyssorum*	*Diaptomus cyaneus*	**<0.001**	**<0.001**	n.s.	**<0.001**	n.s.	n.s.
	*Eudiaptomus vulgaris*	**<0.001**	**<0.001**	**<0.001**	**<0.001**	**<0.001**	
	*Mixodiaptomus laciniatus*	n.s.	**<0.001**	**<0.001**	**<0.001**	**<0.001**	**<0.001**
	*Alona affinis*	**<0.001**	**<0.001**	**0.015**	**<0.001**	**<0.001**	n.s.
	*Daphnia longispina*	**<0.001**	n.s.	**<0.001**	**<0.001**	**<0.001**	n.s.
	*Daphnia pulicaria*	**<0.001**	**<0.001**	**<0.001**	**<0.001**	**<0.001**	n.s.
*Diaptomus cyaneus*	*Eudiaptomus vulgaris*	n.s.	**<0.001**	**<0.001**	n.s.	**<0.001**	
	*Mixodiaptomus laciniatus*	**<0.001**	**<0.001**	**<0.001**	n.s.	**<0.001**	**<0.001**
	*Alona affinis*	n.s.	**<0.001**	n.s.	**<0.001**	**<0.001**	n.s.
	*Daphnia longispina*	**<0.001**	**<0.001**	**<0.001**	**<0.001**	**<0.001**	n.s.
	*Daphnia pulicaria*	n.s.	**<0.001**	**<0.001**	**<0.001**	**<0.001**	**0.026**
*Eudiaptomus vulgaris*	*Mixodiaptomus laciniatus*	**<0.001**	n.s.	n.s.	n.s.	n.s.	
	*Alona affinis*	n.s.	**0.007**	n.s.	**<0.001**	**<0.001**	
	*Daphnia longispina*	**0.040**	**<0.001**	**<0.001**	**<0.001**	**0.001**	
	*Daphnia pulicaria*	n.s.	n.s.	**<0.001**	**<0.001**	n.s.	
*Mixodiaptomus laciniatus*	*Alona affinis*	**0.001**	**<0.001**	n.s.	**<0.001**	**<0.001**	n.s.
	*Daphnia longispina*	**<0.001**	**0.019**	**<0.001**	**<0.001**	n.s.	**0.003**
	*Daphnia pulicaria*	**<0.001**	n.s.	**<0.001**	**<0.001**	**<0.001**	n.s.
*Alona affinis*	*Daphnia longispina*	n.s.	**<0.001**	**<0.001**	**<0.001**	**<0.001**	n.s.
	*Daphnia pulicaria*	n.s.	**0.001**	**<0.001**	**<0.001**	**0.022**	n.s.
*Daphnia longispina*	*Daphnia pulicaria*	**0.002**	**<0.001**	**<0.001**	**0.037**	**<0.001**	n.s.

Significant results (*p*-value <0.05) are indicated in bold; n.s., not significant.

Because of the major contribution of NAs to the total P pool in organisms [11], %P patterns mirrored those for NAs, especially %RNA (Fig. 1). Overall, differences in %P between copepods and cladocerans were not significant (Tables 2, 3; see inset in Fig. 1D). P represented more than 1.5% of the dry weight in *Cyclops abyssorum* and *Diaptomus cyaneus*, higher than in *Mixodiaptomus laciniatus* but not significantly different from that in cladocerans (Fig. 1D; Table 4).

**Figure 2:**
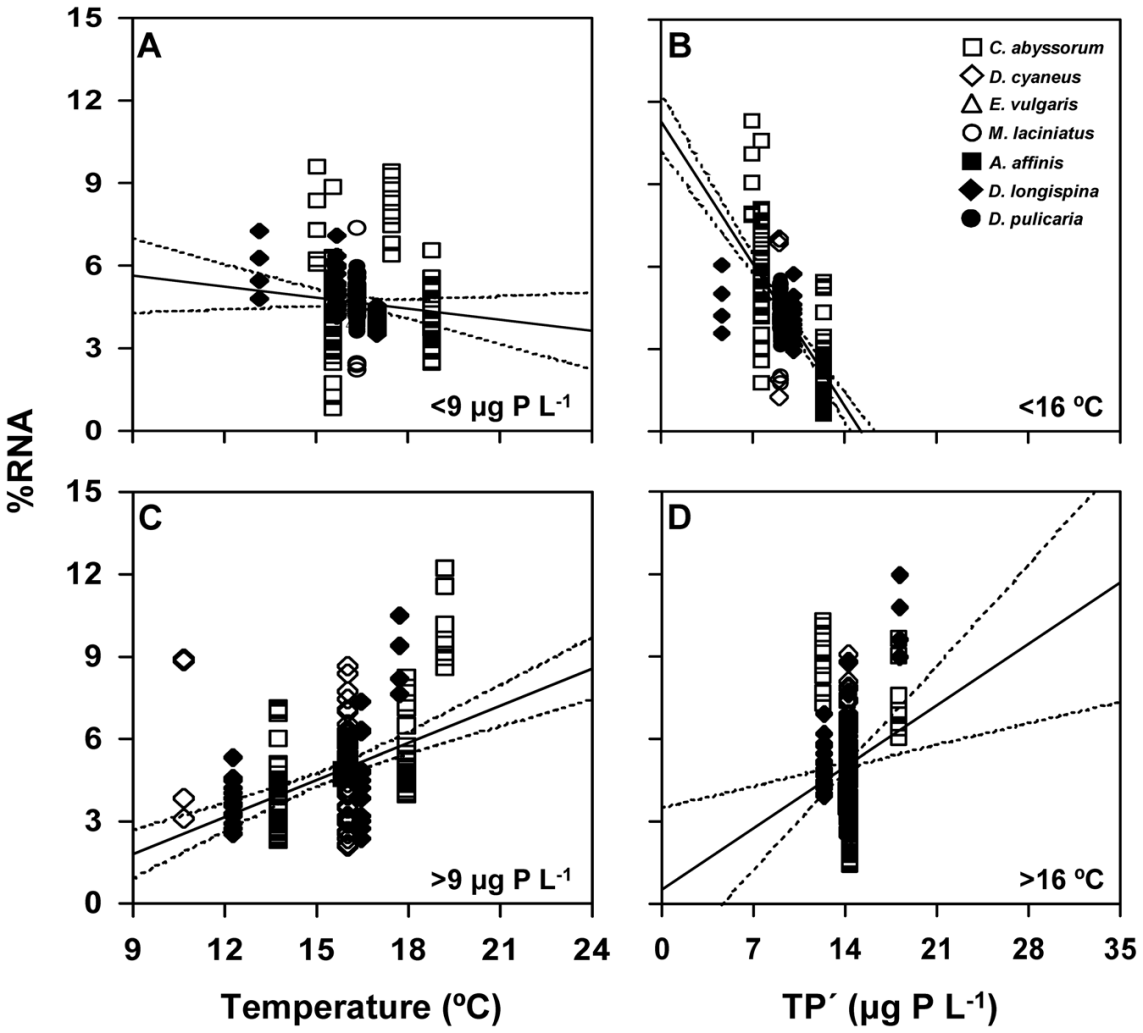
Partial regression plots for the relationships of lake temperature and phosphorus with RNA content for investigated zooplankton species. Relationships of temperature with RNA content (% of dry weight, %RNA) (A) below and (C) above the median value of lake phosphorus (TP′, 9 µg P L^−1^). Relationships of TP′ with %RNA (B) below and (D) above the median value of lake temperature (16°C). Each point represents single observations for each species. Copepod species are *Cyclops (C.) abyssorum*, *Diaptomus (D.) cyaneus*, *Eudiaptomus (E.) vulgaris*, and *Mixodiaptomus (M.) laciniatus*; and cladoceran species are *Alona (A.) affinis*, *Daphnia (D.) longispina*, and *Daphnia (D.) pulicaria*. Dotted lines indicate 95% confidence intervals around the fitted regression lines (solid lines).

Differences among lakes contributed to the observed intergroup and interspecific variability in body size and NAs in zooplankton (Table 2). Temperature, TP′, and their interaction explained a substantial percentage of the variance in body size and most NA variables in zooplankton, especially the variance in %RNA (Table 3). We further examined these effects by splitting our lake data set according to the median values of TP′ and temperature, as depicted in Fig. 2. Antagonistic temperature×TP′ effects were found for %RNA. Thus, the %RNA vs. temperature regressions showed a negative trend below the median TP′ of 9 µg P L^−1^ and a positive one above this value (Fig. 2A vs. 2C, Table 5). Likewise, the %RNA vs. TP′ regressions showed a negative trend below the median temperature of 16°C and a positive one above this value (Fig. 2B vs. 2D, Table 5).

**Table 5:** Results of ANCOVA to analyze intergroup (copepoda vs. cladocera) and interspecific differences, and the single effects of lake temperature below- and above median phosphorus (TP′), and lake TP′ below- and above median temperature on reciprocal square root-transformed RNA content (% of dry weight, %RNA).

Data range	Source of variation	df	*F*	*p*-value
<9 µg P L^−1^	Group	1	0.77	n.s.
	Species (Group)	**4**	**60.99**	**<0.001**
	Temperature	1	2.64	n.s.
	Error	195		
>9 µg P L^−1^	Group	**1**	**211.86**	**<0.001**
	Species (Group)	**2**	**171.04**	**<0.001**
	Temperature	**1**	**59.13**	**<0.001**
	Error	197		
<16°C	Group	**1**	**9.45**	**0.002**
	Species (Group)	**4**	**26.13**	**<0.001**
	TP′	**1**	**103.33**	**<0.001**
	Error	165		
>16°C	Group	**1**	**59.33**	**<0.001**
	Species (Group)	**3**	**210.22**	**<0.001**
	TP′	**1**	**5.40**	**0.021**
	Error	226		

Species (Group) denotes Species nested within Group. Reported are: degrees of freedom (df), *F*-test results (*F*), and significance level (*p*-value). Significant results (*p*-value <0.05) are indicated in bold; n.s., not significant.

### Intraspecific Variability in NAs and P

**Figure 3:**
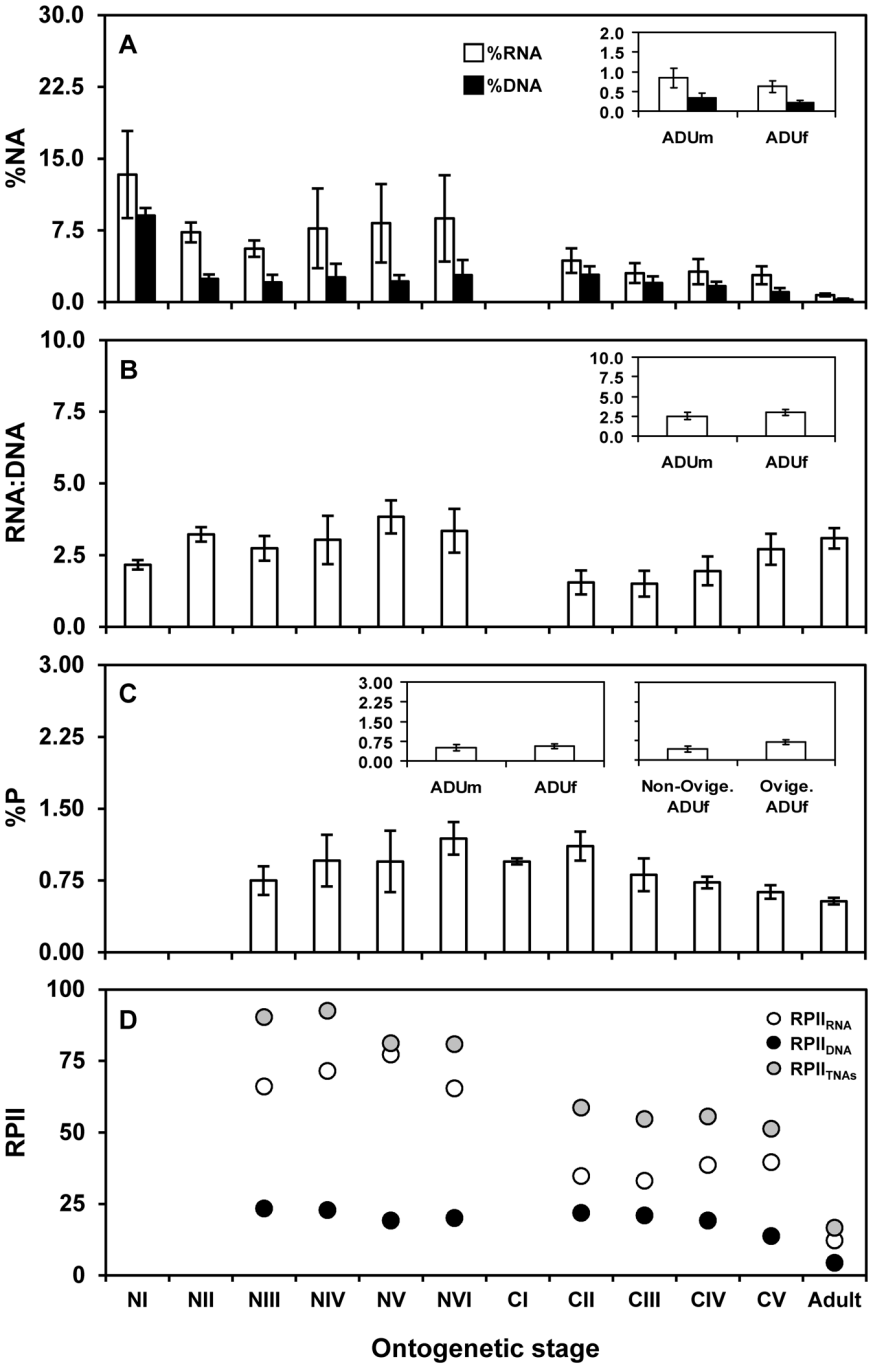
Nucleic acid content, RNA:DNA ratio, total phosphorus content, and relative phosphorus investment indices for the copepod *Mixodiaptomus laciniatus*. (A) Nucleic acid (NA) content (% of dry weight, %NA), (B) RNA:DNA ratio, (C) total phosphorus (P) content (% of dry weight, %P), and (D) relative P investment index (RPII) for RNA (RPII_RNA_), DNA (RPII_DNA_), and total NAs (RPII_TNAs_) of *Mixodiaptomus laciniatus* stages. Insets represent these variables as a function of gender in adulthood and reproductive status in adult females. Columns in A-C and circles in D are mean values. Error bars represent standard deviations for nauplius (NI-NVI), copepodite (CI-CV) and adult (ADUm, adult male; ADUf, adult female; Non-oviger. ADUf, non-ovigerous adult female; Oviger. ADUf, ovigerous adult female) stages.

When possible, we identified ontogeny, gender, and female reproductive status as possible sources of intraspecific variation in NAs for each species and lake combination. The effects of ontogeny varied among species and lakes (Table S2). For example, strong ontogenetic effects were detected for most variables in *Cyclops abyssorum* from lake Estany de la Munyidera, *Diaptomus cyaneus* and *Mixodiaptomus laciniatus*, and no or only minor effects were observed in *Cyclops abyssorum* from lake Estany dels Barbs. Gender influenced %RNA, RNA:DNA ratio, and %P-TNAs in *Diaptomus cyaneus*. The effects of female reproductive status varied widely between *Diaptomus cyaneus* and *Daphnia longispina*, and among *Daphnia longispina* individuals from different lakes, lacking a clear consistent pattern (Table S2).The intraspecific NA variability in the copepod *Mixodiaptomus laciniatus* was examined in a large number of samples (>450 individuals) collected over three years in lake Laguna de la Caldera (2005, 2006, and 2007). A strong effect of both ontogeny and sampling year was observed for all variables (Table 6), with decreasing trends in %RNA, %DNA, %P-TNAs, and %P over the copepod life cycle (Fig. 3; Tables 6, 7). RPII_TNAs_ also decreased during the life cycle (Fig. 3D), as indicated by the significantly negative slope of the linear regression between body size and RPII_TNAs_ (RPII_TNAs_ = −0.072*body size +106.38, *p*-value <0.001, *R^2^* = 0.91). However, RPII_RNA_ was always higher than RPII_DNA_ at each stage, especially at nauplius stages (Fig. 3D). As a consequence, the slope of the regression between body size and RPII_RNA_ (RPII_RNA_ = −0.063*body size +85.04, *p*-value <0.001, *R^2^* = 0.83) was >4-fold higher (ANCOVA: intercept, *F*_1,14_ = 224.80, *p*-value <0.001; slope, *F*_1,14_ = 17.74, *p*-value = 0.001) than the slope between body size and RPII_DNA_ (RPII_DNA_ = −0.015*body size +26.84, *p*-value = 0.012, *R^2^* = 0.61). Because of the tight correlation between body size and DT (body size = 58.82*DT +167.84, *p*-value <0.001, *R^2^* = 0.85; Fig. S2), ontogenetic patterns of RPII using DT as predictor resembled those of body size (RPII_TNAs_ = −2.98*DT +87.32, *p*-value = 0.009, *R^2^* = 0.70; RPII_RNA_ = −2.82*DT +69.07, *p*-value = 0.035, *R^2^* = 0.55; RPII_DNA_ = −0.48*DT +22.83, *p*-value = 0.023, *R^2^* = 0.60). The adults evidenced gender differences in all variables, except for %P; neither the %P differed between ovigerous and non-ovigerous adult females (see insets in Fig. 3A–C; Table 7).

**Table 6:** Results of ANOVA of the effects of ontogeny and sampling year on natural log-transformed body size (µm), RNA and DNA contents (% of dry weight, %RNA and %DNA), RNA:DNA ratio, phosphorus (P) allocated to total nucleic acids (TNAs), and total P content (% of dry weight, %P-TNAs and %P) in the copepod *Mixodiaptomus laciniatus*.

Response variable	Source ofvariation	df	*F*	*p*-value	*PV*
ln(Body size+1)	Ontogeny	**11**	**3264.77**	**<0.001**	**78.41**
	Sampling year	**20**	**36.11**	**<0.001**	**0.16**
	Error	615			
ln(%RNA+1)	Ontogeny	**10**	**178.20**	**<0.001**	**78.15**
	Sampling year	**20**	**110.68**	**<0.001**	**9.71**
	Error	468			
ln(%DNA+1)	Ontogeny	**10**	**90.97**	**<0.001**	**52.73**
	Sampling year	**20**	**26.81**	**<0.001**	**3.11**
	Error	456			
ln(RNA:DNA+1)	Ontogeny	**90**	**11.42**	**<0.001**	**13.82**
	Sampling year	**20**	**82.88**	**<0.001**	**22.28**
	Error	471			
ln(%P-TNAs+1)	Ontogeny	**90**	**185.14**	**<0.001**	**72.45**
	Sampling year	**20**	**66.21**	**<0.001**	**5.76**
	Error	470			
ln(%P+1)	Ontogeny	**90**	**7.32**	**<0.001**	**64.04**
	Sampling year				
	Error	37			

Reported are: degrees of freedom (df), *F*-test results (*F*), significance level (*p*-value), and percentage of variance (*PV*) calculated as (sum of squares of treatment/total sum of squares)×100. Significant results (*p*-value <0.05) are indicated in bold.

**Table 7:** Results of Tukey’s HSD post-hoc tests to analyze differences in natural log-transformed body size (µm), RNA and DNA contents (% of dry weight, %RNA and %DNA), RNA:DNA ratio, phosphorus (P) allocated to total nucleic acids (TNAs), and total P content (% of dry weight, %P-TNAs and %P) between successive ontogenetic stages (NI-NVI, nauplius stages; CI-CV, copepodite stages; adult stage), adult genders (male vs. female), and female reproductive statuses (non-ovigerous vs. ovigerous) in the copepod *Mixodiaptomus laciniatus*.

		ln(Body size+1)	ln(%RNA+1)	ln(%DNA+1)	ln(RNA:DNA+1)	ln(%P-TNAs+1)	ln(%P+1)
		*p*-value	*p*-value	*p*-value	*p*-value	*p*-value	*p*-value
NI	NII	**<0.001**	**0.006**	**<0.001**			
NII	NIII	**<0.001**	**0.042**	n.s.	n.s.	**0.020**	
NIII	NIV	**<0.001**	n.s.	n.s.	n.s.	**0.006**	.n.s.
NIV	NV	**<0.001**	n.s.	n.s.	n.s.	n.s.	.n.s.
NV	NVI	**<0.001**	n.s.	n.s.	n.s.	n.s.	.n.s.
NVI	CI	**<0.001**					.n.s.
CI	CII	**<0.001**					.n.s.
CII	CIII	**<0.001**	**<0.001**	**<0.001**	n.s.	**<0.001**	.n.s.
CIII	CIV	**<0.001**	n.s.	n.s.	n.s.	n.s.	.n.s.
CIV	CV	**<0.001**	n.s.	**<0.001**	**<0.001**	**<0.001**	.n.s.
CV	Adult	**<0.001**	**<0.001**	**<0.001**	n.s.	**<0.001**	.n.s.
Male	Female	**<0.001**	**<0.001**	**<0.001**	**0.018**	**<0.001**	.n.s.
Non-ovigerous female	Ovigerous female						.n.s.

Significant results (*p*-value <0.05) are indicated in bold; n.s., not significant.

### Sources of NA Variation

**Figure 4:**
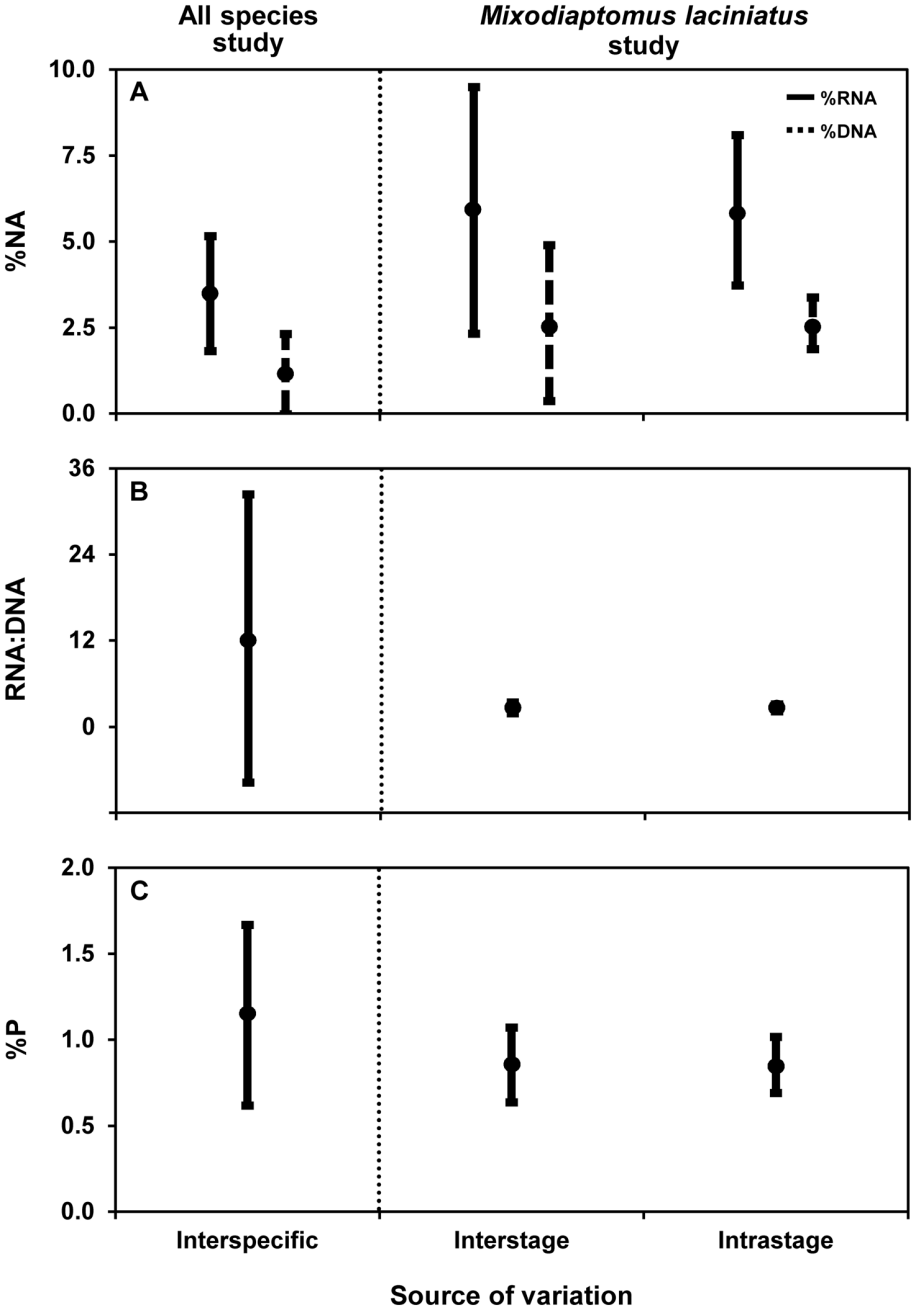
Inter- and intraspecific variabilities in nucleic acid content, RNA:DNA ratio, and total phosphorus content. The diagram illustrates the variability (error bars) in (A) nucleic acid (NA) content (% of dry weight, %NA), (B) RNA:DNA ratio, and (C) total phosphorus (P) content (% of dry weight, %P). Interspecific and interstage variabilities were obtained by calculating the standard deviation of means for all species and stages of the copepod *Mixodiaptomus laciniatus*, respectively. Intrastage variability for *Mixodiaptomus laciniatus* was obtained by calculating mean stage-specific standard deviations. Circles are mean values.

The sources of NA variation were elucidated by examining the variability in NA and P contents among all species (interspecific) and among (interstage) and within (intrastage) developmental stages of *Mixodiaptomus laciniatus* (Fig. 4). Intraspecific variability contributed most to the %RNA variability in *Mixodiaptomus laciniatus*, whereas the RNA:DNA ratio variability was highest among species and relatively low within them (Fig. 4).

### Testing the GRH

**Figure 5:**
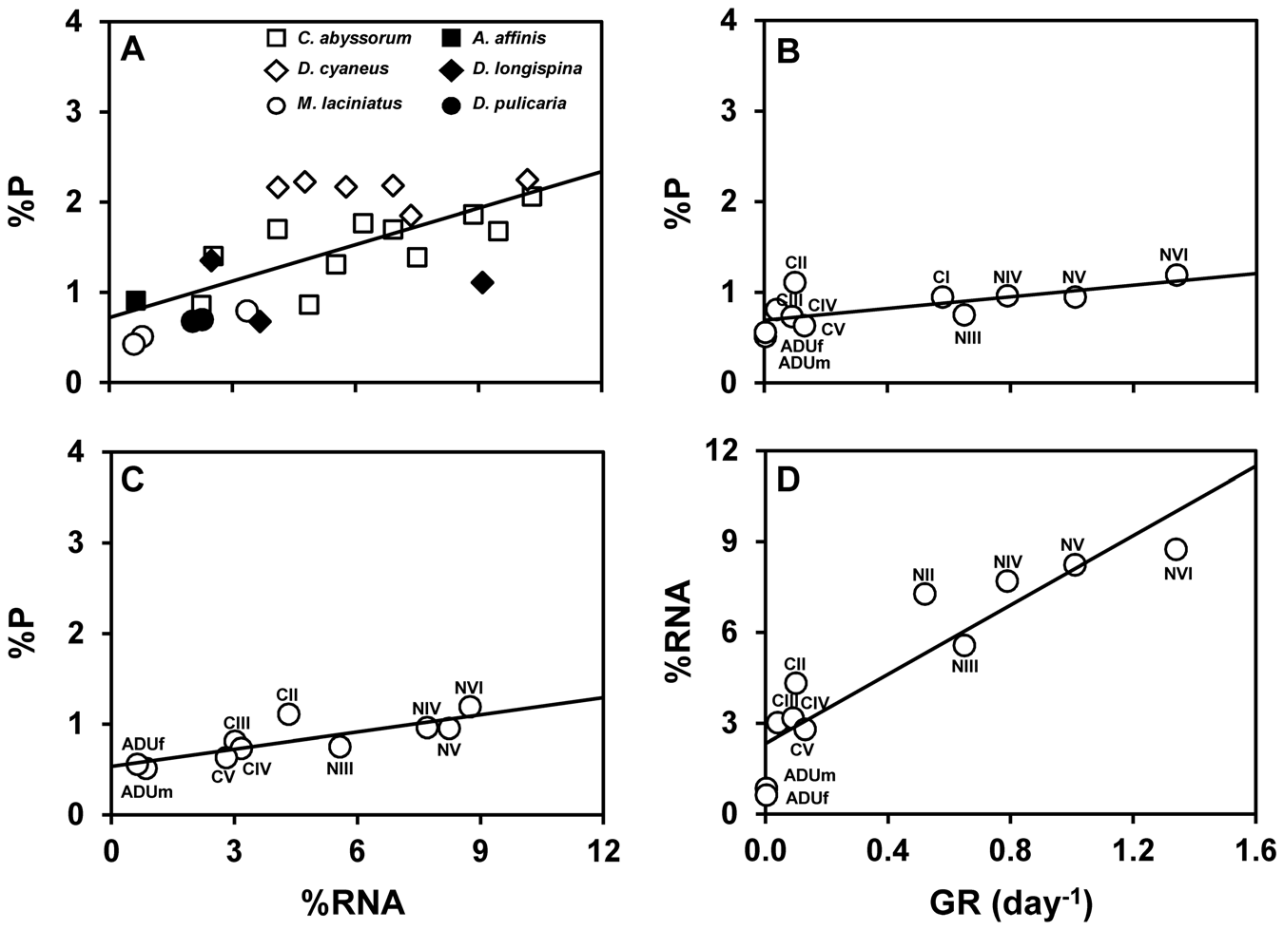
Growth rate hypothesis relationships for all species and *Mixodiaptomus laciniatus* studies. Relationships of RNA (% of dry weight, %RNA) with total phosphorus (P) content (% of dry weight, %P) for (A) all crustacean species in this study and (C) all ontogenetic stages of the copepod *Mixodiaptomus laciniatus* from lake Laguna de la Caldera; and relationships of growth rate (GR) with (B) %P, and (D) %RNA for *Mixodiaptomus laciniatus* stages. Each point represents mean values for each species×stage×lake combination in panel A, or ontogenetic stage in panels B–D. Copepod species are *Cyclops (C.) abyssorum*, *Diaptomus (D.) cyaneus*, and *Mixodiaptomus (M.) laciniatus*; and cladoceran species are *Alona (A.) affinis*, *Daphnia (D.) longispina*, and *Daphnia (D.) pulicaria*. Stages are nauplii (NI-NVI), copepodites (CI-CV), and adults (ADUm, adult male; ADUf, adult female). Solid lines are linear regression fits. See Table 8 for regression parameters and statistics.

A close covariation was found between %RNA and %P (Fig. 5A; Table 8) in all crustacean species, based on the mean values for the different ontogenetic stages and genders (copepods) and for the non-ovigerous and ovigerous females (copepods and cladocerans) from different lakes. We also observed strong positive linear regressions (%P vs. GR, %P vs. %RNA, %RNA vs. GR) for the different ontogenetic stages of *Mixodiaptomus laciniatus* (Fig. 5B–D; Table 8).

**Table 8:** Results of linear regression analyses to test the growth rate hypothesis [5], [11], [12].

	Dependent variable	Independent variable	Intercept	Slope	*p*-value	*R^2^*
All species	%P	%RNA	**0.72**	**0.13**	**<0.001**	**0.46**
*Mixodiaptomus laciniatus*	%P	GR	**0.70**	**0.32**	**00.020**	**0.47**
	%P	%RNA	**0.54**	**0.06**	**00.004**	**0.67**
	%RNA	GR	**2.32**	**5.73**	**<0.001**	**0.84**

Variables: GR, growth rate (days^−1^); %RNA, mean RNA content (% of dry weight); and %P, mean total phosphorus content (% of dry weight). Reported are: intercept, slope, significance level (*p*-level) and coefficient of determination (*R^2^*). Significant results (*p*-value <0.05) are indicated in bold.

## Discussion

Our results for 22 high mountain lakes support the role of phylogeny, life history strategies, and environmental (temperature and nutrient) constraints on the NA content of zooplankton, illustrating how the integration of MTE and BS principles can successfully explain fundamental processes at the organism level.

RNA:DNA ratios were higher in cladocerans than in copepods, as previously reported, consistent with the PAH premise that the allocation of P from DNA to RNA may drive elevated RNA:DNA ratios and therefore a reduced genome size in cladocerans [13]. However, our results call into question the categorical designation of copepods as ‘slow-growth' organisms with low-P, -RNA, and high DNA and of cladocerans as ‘fast-growth' organisms with high-P, -RNA, and low DNA. This is because a considerable variation in the NA content of selected cladoceran and copepod taxa was found over a wide range of systems, regardless of their taxonomic affiliation. For example, the reduced RNA and RNA:DNA ratio and elevated DNA in *Alona affinis* were more similar to the NA profile of *Mixodiaptomus laciniatus* or *Eudiaptomus vulgaris* than to that of the phylogenetically closer *Daphnia* species. Furthermore, the RNA and P content was markedly higher in *Cyclops abyssorum* or *Diaptomus cyaneus* than in *Mixodiaptomus laciniatus* or *Daphnia* species. Hence, we observed a pronounced taxa-specific variation in NA composition within each group (copepods and cladocerans).

We propose that much of the variation in NAs observed may also be attributed to life-history strategies. Thus, while *Diaptomus cyaneus* is often associated with non-permanent water bodies and completes its life cycle within 3–4 weeks [45], [46], *Mixodiaptomus laciniatus* inhabits more permanent water bodies and typically completes an univoltine cycle in alpine systems within 4–5 months [14], [47], [48]. These systematic differences may reflect distinct selective pressures from P requirements for growth. Whereas *Mixodiaptomus laciniatus* feeds on scarce pelagic high quality seston [25], [49], [50], the high P-requirements of *Diaptomus cyaneus* may be met by omnivorous feeding [30] and by exploiting both littoral and benthic environments [49]. Likewise, the higher levels of NAs and P in copepodites than nauplii for *Cyclops abyssorum* might be associated with the switch from an herbivorous diet in nauplii to a carnivorous diet based on high-P *Daphnia* in copepodites at metamorphosis [51]. Lastly, exploitation of benthic P-enriched resources and a microphagous feeding mode [52], [53], [54] may also allow *Alona affinis* to overcome P-limitation and maintain a large genome in comparison to *Daphnia* species [55].

Previous research on *Daphnia* has also indicated that NA variability may be attributable to ontogenetic variation [56]. Our results add to previous observations of marked intraspecific changes in the NA content and RNA:DNA ratio of marine calanoid copepod species [57], with a general decrease in both %RNA and %DNA from nauplius to adult stages. We also contribute evidence that the magnitude of ontogenetic variation in *Mixodiaptomus laciniatus,* especially for RNA, is comparable to or larger than the variation with other zooplankton species. The decrease in %RNA up to nauplius stage III may be explained by the depletion of inherited maternal RNA for early protein synthesis [48]. Immediately afterwards, nauplii become self-feeding and show an increase in RNA synthesis, which possibly results from the higher protein demand for growth, cellular proliferation, and differentiation before metamorphosis [57]. Although the relative NA content remained relatively low after metamorphosis, a further decrease was observed in adulthood, possibly attributable to the major increase in weight due to lipid storage [19]. This pattern is consistent with the ontogenetical decrease in RPII_TNAs_, which reflects a reduction in P allocation to NAs towards adulthood, which may be linked to an increase in other P-enriched biomolecules [11].

However, the intraspecific variability in NAs was not restricted to interstage shifts, given that strong changes in RNA were also observed within *Mixodiaptomus laciniatus* stages (intrastage variability) (see Fig. 4A). This corresponds well with observations of major variations in biochemical and elemental constituents at stage level in zooplankton [14], [19], [58]. Carrillo et al. [14] described large intrastage variations in the P content of *Mixodiaptomus laciniatus* with a trend towards a greater P content as individuals in inter- and premolt phases (active cellular division) approach the ecdysis associated with a molt event (see Figs. 3 and 6 in [14]). Alternatively, it was recently proposed that intrastage differences in RNA may be attributed to varying within-stage sensitivity to food quality for animal growth [59].

Our results also showed strong relationships between the contents of RNA and P across studied species of mesozooplankton and between these contents and GR for the developmental stages of *Mixodiaptomus laciniatus*. Trends were all consistent with GRH predictions [5], [12]. A notable finding was that regression slopes between GR and %P and between %RNA and %P were considerably lower for *Mixodiaptomus laciniatus* in this study (0.32 and 0.06, respectively) than those reported by Elser et al. [12] for the cladocerans *Daphnia pulicaria* (1.32 and 1.69, respectively) and *Daphnia galeata* (0.73 and 1.78, respectively). These variations likely reflect interspecific differences in life-history strategies between ‘fast-growth’ *Daphnia* species (like *r*-strategists) and ‘slow-growth’ copepod species (like *K*-strategists).

However, the finding that NA values in the species were influenced not only by phylo- and ontogenetic constraints but also by lake of origin, is consistent with the notion that other factors may have an important impact on the NA content of organisms. Many relevant parameters might vary among lakes. In particular, the opposed effects of temperature on RNA content under distinct nutrient scenarios contrast with the well-established positive effect of temperature on metabolic rate as predicted by MTE [2], [3]. It has been reported that recent temperature changes have already impacted organisms in multiple ways [60]. However, our results find evidence of more subtle effects of temperature on organisms via GR-RNA-P couplings that depend on the trophic status of the ecosystem. Thus, the inhibitory effect of temperature on RNA at low nutrient conditions suggests that organisms growing in oligotrophic systems might be particularly vulnerable to global warming. Another major environmental perturbation is the eutrophication of freshwater and marine ecosystems [61]. The present observation of a temperature-dependent role of nutrients on RNA challenges the ‘common sense' prediction in ecology that higher resource availability for autotrophs should increase primary production, and thereby stimulate consumer growth (e.g. [62], [63]). The observed detrimental effect of nutrients on the growth of organisms in cold waters is consistent with experimental and natural observations of weakened consumer growth after nutrient enrichments [50], [64]. While recent work has emphasized the role of aquatic systems as ‘sentinels of climate change' [65], not all types of lakes might give clear signs of the impact of a particular climatic stressor due to the numerous confounding factors that can affect lakes and their catchment areas [66]. Our finding that the response of organism growth and NAs differed from MTE- and BS-predicted patterns at low temperature and nutrient environments highlight the ultra-sensitivity of alpine lake ecosystems to shifts in climate, which is consistent with previous research results [67], [68].

Taken together, our results are consistent with stoichiometric predictions (PAH and GRH) for NAs in crustacean zooplankton. However, RNA and DNA content did not evidence a consistent pattern of species-phylogenetic affiliation to cladocerans or copepods. Therefore, the PAH needs to take into account other important mechanisms, including life-history strategies, ontogenetic variations, and their temperature- and nutrient-dependence. We highlight the observation that the interaction between temperature and TP′ accounts for a substantial part of the variability of NAs in zooplankton, because it provides empirical evidence for the mechanistic MTE and BS-principles underpinning NAs composition and therefore advances our progress towards a more synthetic theory of Ecology.

## Supporting Information

Figure S1 **Zooplankton biomass and taxonomic composition of the study lakes.** (A) Biomass and taxonomic composition of the zooplankton, and (B) % of total zooplankton biomass for each taxonomic group of the study lakes: *Acanthocyclops (A.) vernalis*, *Cyclops (C.) abyssorum*, *Diaptomus (D.) cyaneus*, *Eudiaptomus (E.) vulgaris*, *Mixodiaptomus (M.) laciniatus*, *Alona (A.) affinis*, *Alona* sp., *Chydorus (C.) sphaericus*, *Daphnia (D.) longispina*, *Daphnia (D.) pulicaria*, Rotifera (rotifers) and Ciliata (ciliates). Lakes are: Caballo, Laguna del Caballo; Yeguas, Laguna de las Yeguas; Gr-Virgen, Lagunillo Grande de la Virgen; Ch-Virgen, Lagunillo Chico de la Virgen; A-Verdes, Laguna de Aguas Verdes; Al-Río Seco, Laguna Alta de Río Seco; Gr-Río Seco, Laguna Grande de Río Seco; Larga, Laguna Larga; Caldera, Laguna de la Caldera; Caldereta, Laguna de la Caldereta; Borreguil, Laguna del Borreguil; Llebreta, Estany de Llebreta; Al-Mont, Estany Alt de Montcasau; Llong, Estany Llong; Redó, Estany Redó; Barbs, Estany dels Barbs; Coveta, Estany de la Coveta; and Cabana, Estany de la Cabana. Lakes with zooplankton biomass values <1 µg dry weight L^−1^ (Laguna de la Gabata, Laguna Hondera, Estany Baix de Montcasau, and Estany de la Munyidera) were excluded.
(PDF)

Figure S2 **Relationship between developmental time and mean body size for ontogenetic stages of the copepod** ***Mixodiaptomus laciniatus*** **.** Stages are nauplii (NI-NVI) and copepodites (CI–CV). Solid line is the linear regression fit. See Results in the main text for regression parameters and statistics.
(PDF)

Table S1 Characterization of high-mountain lakes in Sierra Nevada and the Pyrenees during the study period. Variables: latitude; longitude; altitude; perimeter; area; maximum depth; *K_d__UVR_*, mean extinction coefficient for ultraviolet radiation (UVR) of 305, 320, and 380 nm; *K_d__PAR_*, extinction coefficient for photosynthetic active radiation (PAR); temp., temperature. Units are given in brackets.
(PDF)

Table S2 Results of ANOVAs to analyze differences in reciprocal square root-transformed body size (µm), RNA and DNA contents (% of dry weight, %RNA and %DNA), RNA:DNA ratio, and phosphorus allocated to total nucleic acids (% of dry weight, %P-TNAs) among ontogenetic stages (nauplius vs. copepodite vs. adult) and between adult genders (male vs. female) for copepods (*Cyclops abyssorum*, *Diaptomus cyaneus*, *Mixodiaptomus laciniatus*), and between female reproductive statuses (non-ovigerous vs. ovigerous) for copepods and cladocerans (*Daphnia longispina*). Significant results (*p*-value <0.05) are indicated in bold; n.s., not significant.
(PDF)
