# Supplementary material for: Nucleic Acid Content in Crustacean Zooplankton: Bridging Metabolic and Stoichiometric Predictions
Source: PLoS One. 2014 Jan 21;9(1):e86493. doi: 10.1371/journal.pone.0086493 (PMC3897710; doi:10.1371/journal.pone.0086493)
Supplement: Figure S1 — Zooplankton biomass and taxonomic composition of the study lakes. (A) Biomass and taxonomic composition of the zooplankton, and (B) % of total zooplankton biomass for each taxonomic group of the study lakes: Acanthocyclops (A.) vernalis, Cyclops (C.) abyssorum, Diaptomus (D.) cyaneus, Eudiaptomus (E.) vulgaris, Mixodiaptomus (M.) laciniatus, Alona (A.) affinis, Alona sp., Chydorus (C.) sphaericus, Daphnia (D.) longispina, Daphnia (D.) pulicaria, Rotifera (rotifers) and Ciliata (ciliates). Lakes are: Caballo, Laguna del Caballo; Yeguas, Laguna de las Yeguas; Gr-Virgen, Lagunillo Grande de la Virgen; Ch-Virgen, Lagunillo Chico de la Virgen; A-Verdes, Laguna de Aguas Verdes; Al-Río Seco, Laguna Alta de Río Seco; Gr-Río Seco, Laguna Grande de Río Seco; Larga, Laguna Larga; Caldera, Laguna de la Caldera; Caldereta, Laguna de la Caldereta; Borreguil, Laguna del Borreguil; Llebreta, Estany de Llebreta; Al-Mont, Estany Alt de Montcasau; Llong, Estany Llong; Redó, Estany Redó; Barbs, Estany dels Barbs; Coveta, Estany de la Coveta; and Cabana, Estany de la Cabana. Lakes with zooplankton biomass values <1 µg dry weight L−1 (Laguna de la Gabata, Laguna Hondera, Estany Baix de Montcasau, and Estany de la Munyidera) were excluded. (PDF) [file pone.0086493.s001.pdf]

Figure S1. Zooplankton biomass and taxonomic composition of the study lakes. (A) Biomass and taxonomic composition of the zooplankton, and (B) % of total zooplankton biomass for each taxonomic group of the study lakes: *Acanthocyclops (A.) vernalis*, *Cyclops (C.) abyssorum*, *Diaptomus (D.) cyaneus*, *Eudiaptomus (E.) vulgaris*, *Mixodiaptomus (M.) laciniatus*, *Alona (A.) affinis*, *Alona* sp., *Chydorus (C.) sphaericus*, *Daphnia (D.) longispina*, *Daphnia (D.) pulicaria*, Rotifera (rotifers) and Ciliata (ciliates). Lakes are: Caballo, Laguna del Caballo; Yeguas, Laguna de las Yeguas; Gr-Virgen, Lagunillo Grande de la Virgen; Ch-Virgen, Lagunillo Chico de la Virgen; A-Verdes, Laguna de Aguas Verdes; Al-Río Seco, Laguna Alta de Río Seco; Gr-Río Seco, Laguna Grande de Río Seco; Larga, Laguna Larga; Caldera, Laguna de la Caldera; Caldereta, Laguna de la Caldereta; Borreguil, Laguna del Borreguil; Llebreta, Estany de Llebreta; Al-Mont, Estany Alt de Montcasau; Llong, Estany Llong; Redó, Estany Redó; Barbs, Estany dels Barbs; Coveta, Estany de la Coveta; and Cabana, Estany de la Cabana. Lakes with zooplankton biomass values <1 µg dry weight L^{-1} (Laguna de la Gabata, Laguna Hondera, Estany Baix de Montcasau, and Estany de la Munyidera) were excluded.

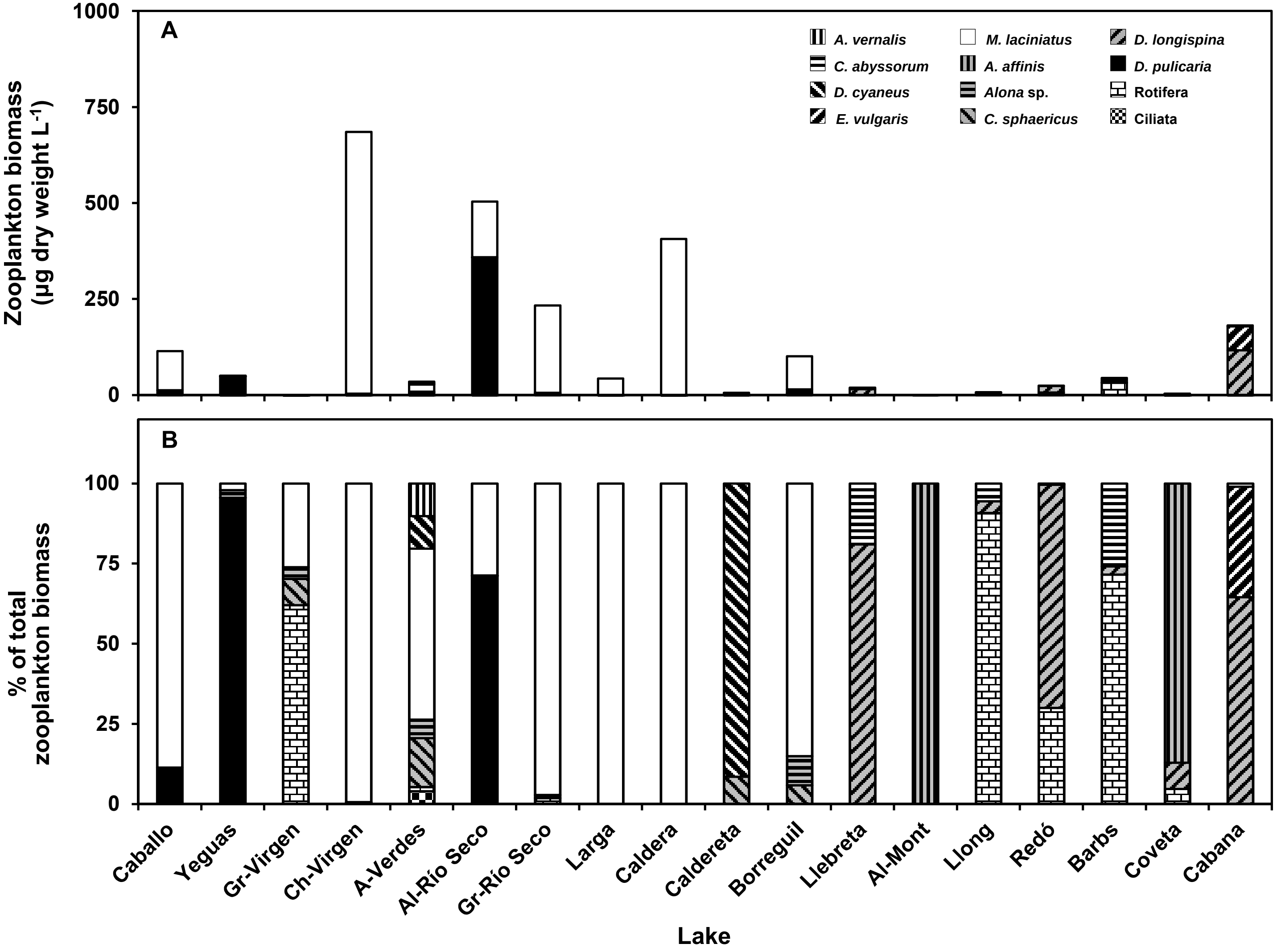
A
B
Zooplankton biomass (µg dry weight L-1)
% of total zooplankton biomass
1000
750
500
250
0
100
75
50
25
0
A. vernalis
C. abyssorum
D. cyaneus
E. vulgaris
M. laciniatus
A. affinis
Alona sp.
C. sphaericus
D. longispina
D. pulicaria
Rotifera
Ciliata
Caballo
Yeguas
Gr-Virgen
Ch-Virgen
A-Verdes
Al-Río Seco
Gr-Río Seco
Larga
Caldera
Caldereta
Borreguil
Llebreta
Al-Mont
Llong
Redó
Barbs
Coveta
Cabana
Lake
