# Supplementary material for: Nucleic Acid Content in Crustacean Zooplankton: Bridging Metabolic and Stoichiometric Predictions
Source: PLoS One. 2014 Jan 21;9(1):e86493. doi: 10.1371/journal.pone.0086493 (PMC3897710; doi:10.1371/journal.pone.0086493)

Figure S2. Relationship between developmental time and mean body size for ontogenetic stages of the copepod *Mixodiaptomus laciniatus*. Stages are nauplii (NI-NVI) and copepodites (CI-CV). Solid line is the linear regression fit. See Results in the main text for regression parameters and statistics.

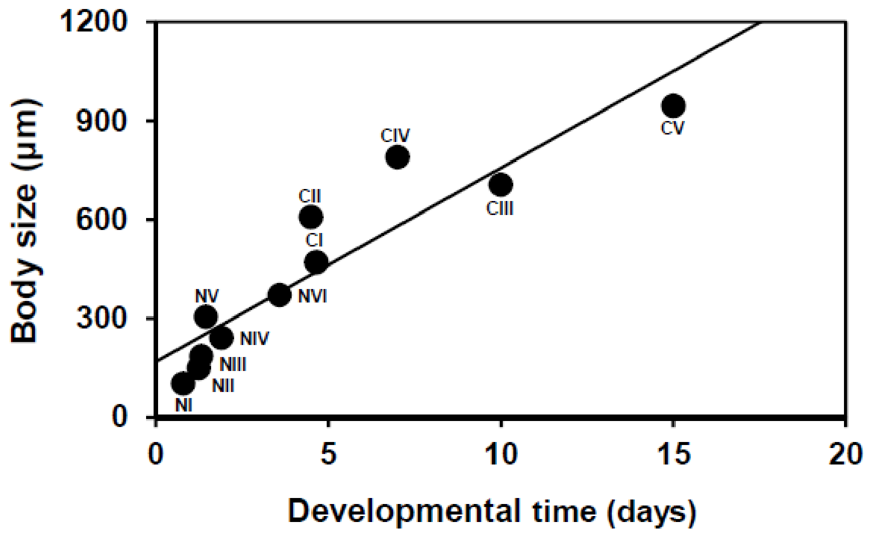

Supplement: Figure S2 — Relationship between developmental time and mean body size for ontogenetic stages of the copepod Mixodiaptomus laciniatus . Stages are nauplii (NI-NVI) and copepodites (CI–CV). Solid line is the linear regression fit. See Results in the main text for regression parameters and statistics. (PDF) [file pone.0086493.s002.pdf]
