# Supplementary material for: Nucleic Acid Content in Crustacean Zooplankton: Bridging Metabolic and Stoichiometric Predictions
Source: PLoS One. 2014 Jan 21;9(1):e86493. doi: 10.1371/journal.pone.0086493 (PMC3897710; doi:10.1371/journal.pone.0086493)
Supplement: Table S1 — Characterization of high-mountain lakes in Sierra Nevada and the Pyrenees during the study period. Variables: latitude; longitude; altitude; perimeter; area; maximum depth; KdUVR, mean extinction coefficient for ultraviolet radiation (UVR) of 305, 320, and 380 nm; KdPAR, extinction coefficient for photosynthetic active radiation (PAR); temp., temperature. Units are given in brackets. (PDF) [file pone.0086493.s003.pdf]

Table S1. Characterization of high-mountain lakes in Sierra Nevada and the Pyrenees during the study period.

Lake	Mountain region	Latitude	Longitude	Altitude (m a.s.l.)	Perimeter (m)	Area (ha)	Maximum depth (m)	Water body type	$K_{d\ UVR}$ (m^{-1})	$K_{d\ PAR}$ (m^{-1})	Temp. (ºC)
Laguna del Caballo	Sierra Nevada	37º00´53.12´´N	3º26´15.71´´W	2851	309.13	0.53	1.97	Permanent	5.75	0.64	14.96
Laguna de las Yeguas	Sierra Nevada	37º03´21.91´´N	3º22´50.81´´W	2886	775.13	3.10	5.45	Permanent	1.79	0.54	15.20
Lagunillo Grande de la Virgen	Sierra Nevada	37º03´02.65´´N	3º22´47.91´´W	2955	290.28	0.55	0.77	Temporary	1.19	0.45	11.83
Lagunillo Chico de la Virgen	Sierra Nevada	37º03´07.00´´N	3º22´46.83´´W	2949	155.73	0.09	0.29	Temporary	7.12	0.94	20.26
Laguna de Aguas Verdes	Sierra Nevada	37º02´54.75´´N	3º22´06.15´´W	3067	273.36	0.36	1.22	Permanent	7.11	1.05	16.23
Laguna Alta de Río Seco	Sierra Nevada	37º03´06.81´´N	3º20´53.30´´W	3052	183.28	0.14	1.28	Permanent	4.35	1.82	17.10
Laguna Grande de Río Seco	Sierra Nevada	37º03´07.86´´N	3º20´44.49´´W	3032	535.84	0.95	1.42	Permanent	4.07	2.66	15.46
Laguna de la Gabata	Sierra Nevada	37º03´36.20´´N	3º20´13.07´´W	2786	228.58	0.19	1.93	Permanent	0.92	0.38	12.21
Laguna Larga	Sierra Nevada	37º03´34.45´´N	3º20´03.59´´W	2789	714.47	2.35	3.38	Permanent	0.37	0.20	16.06
Laguna de la Caldera	Sierra Nevada	37º03´17.66´´N	3º19´44.85´´W	3030	499.81	1.81	1.48	Permanent	2.49	0.54	14.87
Laguna de la Caldereta	Sierra Nevada	37º03´12.64´´N	3º19´27.30´´W	3045	230.31	0.35	0.92	Temporary	2.02	0.66	17.64
Laguna del Borreguil	Sierra Nevada	37º03´09.84´´N	3º17´59.24´´W	2983	180.85	0.22	1.09	Permanent	3.74	0.82	16.42
Laguna Hondera	Sierra Nevada	37º02´52.96´´N	3º17´39.55´´W	2899	1113.02	3.90	0.21	Permanent	3.49	1.60	14.50
Estany de Llebreta	Pyrenees	42º32´59.16´´N	0º53´19.08´´E	1620	1664.66	7.22	4.63	Permanent	2.31	0.36	16.47
Estany Baix de Montcasau	Pyrenees	42º38´26.83´´N	0º54´06.65´´E	2039	688.82	1.96	4.95	Permanent	1.47	0.24	14.89
Estany Alt de Montcasau	Pyrenees	42º38´20.34´´N	0º54´17.49´´E	2049	688.40	2.01	5.31	Permanent	1.60	0.30	14.01
Estany Llong	Pyrenees	42º34´23.11´´N	0º57´00.39´´E	2000	1979.11	7.59	5.79	Permanent	1.33	0.27	17.37
Estany Redó	Pyrenees	42º34´51.66´´N	0º57´30.57´´E	2116	1010.80	6.34	6.87	Permanent	0.67	0.20	16.02
Estany dels Barbs	Pyrenees	42º35´57.00´´N	0º58´50.09´´E	2378	899.76	2.47	12.75	Permanent	0.96	0.44	12.27
Estany de la Munyidera	Pyrenees	42º36´02.23´´N	0º58´50.32´´E	2369	746.75	0.91	5.62	Permanent	0.68	0.13	15.43
Estany de la Coveta	Pyrenees	42º32´34.59´´N	1º02´08.06´´E	2399	721.00	2.03	5.47	Permanent	1.21	0.29	17.70
Estany de la Cabana	Pyrenees	42º32´52.15´´N	1º02´18.83´´E	2379	690.13	2.20	5.85	Permanent	2.03	0.34	18.67

Variables: latitude; longitude; altitude; perimeter; area; maximum depth; $K_{d\ UVR}$, mean extinction coefficient for ultraviolet radiation (UVR) of 305, 320, and 380 nm; $K_{d\ PAR}$, extinction coefficient for photosynthetic active radiation (PAR); temp., temperature. Units are given in brackets.
