# Supplementary material for: Nucleic Acid Content in Crustacean Zooplankton: Bridging Metabolic and Stoichiometric Predictions
Source: PLoS One. 2014 Jan 21;9(1):e86493. doi: 10.1371/journal.pone.0086493 (PMC3897710; doi:10.1371/journal.pone.0086493)
Supplement: Table S2 — Results of ANOVAs to analyze differences in reciprocal square root-transformed body size (µm), RNA and DNA contents (% of dry weight, %RNA and %DNA), RNA:DNA ratio, and phosphorus allocated to total nucleic acids (% of dry weight, %P-TNAs) among ontogenetic stages (nauplius vs. copepodite vs. adult) and between adult genders (male vs. female) for copepods (Cyclops abyssorum, Diaptomus cyaneus, Mixodiaptomus laciniatus), and between female reproductive statuses (non-ovigerous vs. ovigerous) for copepods and cladocerans (Daphnia longispina). Significant results (p-value <0.05) are indicated in bold; n.s., not significant. (PDF) [file pone.0086493.s004.pdf]

Table S2. Results of ANOVAs to analyze differences in reciprocal square root-transformed body size (µm), RNA and DNA contents (% of dry weight, %RNA and %DNA), RNA:DNA ratio, and phosphorus allocated to total nucleic acids (% of dry weight, %P-TNAs) among ontogenetic stages (nauplius vs. copepodite vs. adult) and between adult genders (male vs. female) for copepods (*Cyclops abyssorum*, *Diaptomus cyaneus*, *Mixodiaptomus laciniatus*), and between female reproductive statuses (non-ovigerous vs. ovigerous) for copepods and cladocerans (*Daphnia longispina*).

Species	Lake	Response variable	Ontogeny				Adult gender				Female reproductive status			
			df_1	df_2	*F*	*p*-value	df_1	df_2	*F*	*p*-value	df_1	df_2	*F*	*p*-value
		$1/\sqrt{\text{Body size}}$	**1**	**55**	**13.87**	**<0.001**								
		$1/\sqrt{\%\text{RNA}}$	1	49	0.01	n.s.								
Cyclops abyssorum	Estany dels Barbs	$1/\sqrt{\%\text{DNA}}$	1	38	0.95	n.s.								
		$1/\sqrt{\text{RNA : DNA}}$	1	37	3.99	n.s.								
		$1/\sqrt{\%\text{P}-\text{TNAs}}$	1	37	0.38	n.s.								
		$1/\sqrt{\text{Body size}}$	**2**	**99**	**128.51**	**<0.001**								
		$1/\sqrt{\%\text{RNA}}$	**2**	**48**	**11.61**	**<0.001**								
Cyclops abyssorum	Estany de la Munyidera	$1/\sqrt{\%\text{DNA}}$	**2**	**43**	**15.02**	**<0.001**								
		$1/\sqrt{\text{RNA : DNA}}$	2	33	1.18	n.s.								
		$1/\sqrt{\%\text{P}-\text{TNAs}}$	**2**	**33**	**10.85**	**<0.001**								
		$1/\sqrt{\text{Body size}}$	**1**	**88**	**269.72**	**<0.001**	1	60	0.69	n.s.	1	42	0.89	n.s.
		$1/\sqrt{\%\text{RNA}}$	**1**	**79**	**24.91**	**<0.001**	**1**	**58**	**72.13**	**<0.001**	**1**	**40**	**10.86**	**0.002**
Diaptomus cyaneus	Laguna de la Caldereta	$1/\sqrt{\%\text{DNA}}$	**1**	**79**	**48.01**	**<0.001**	1	58	1.09	n.s.	**1**	**40**	**14.27**	**0.001**
		$1/\sqrt{\text{RNA : DNA}}$	1	79	0.00	n.s.	**1**	**58**	**87.51**	**<0.001**	1	40	0.08	n.s.
		$1/\sqrt{\%\text{P}-\text{TNAs}}$	**1**	**79**	**32.02**	**<0.001**	**1**	**58**	**51.06**	**<0.001**	**1**	**40**	**12.79**	**0.001**
		$1/\sqrt{\text{Body size}}$	**1**	**81**	**8.29**	**0.005**								
		$1/\sqrt{\%\text{RNA}}$	**1**	**23**	**324.76**	**<0.001**								
Mixodiaptomus laciniatus	Laguna de la Caldera	$1/\sqrt{\%\text{DNA}}$	**1**	**57**	**48.63**	**<0.001**								
		$1/\sqrt{\text{RNA : DNA}}$	1	20	0.31	n.s.								
		$1/\sqrt{\%\text{P}-\text{TNAs}}$	**1**	**20**	**151.94**	**<0.001**								

Significant results (*p*-value < 0.05) are indicated in bold; n.s., not significant.

Species	Lake	Response variable	Ontogeny				Adult gender				Female reproductive status			
			df_1	df_2	*F*	*p*-value	df_1	df_2	*F*	*p*-value	df_1	df_2	*F*	*p*-value
Daphnia longispina	Estany de Llebreta	$1/\sqrt{\text{Body size}}$									**1**	**42**	**7.15**	**0.011**
		$1/\sqrt{\%RNA}$									**1**	**13**	**33.75**	**<0.001**
		$1/\sqrt{\%DNA}$									1	15	3.21	n.s.
		$1/\sqrt{RNA:DNA}$									1	11	2.26	n.s.
		$1/\sqrt{\%P-TNAs}$									**1**	**11**	**39.25**	**<0.001**
Daphnia longispina	Estany Llong	$1/\sqrt{\text{Body size}}$									1	30	3.24	n.s.
		$1/\sqrt{\%RNA}$									1	26	0.00	n.s.
		$1/\sqrt{\%DNA}$									**1**	**23**	**12.45**	**0.002**
		$1/\sqrt{RNA:DNA}$									**1**	**23**	**18.34**	**<0.001**
		$1/\sqrt{\%P-TNAs}$									1	23	0.17	n.s.
Daphnia longispina	Estany dels Barbs	$1/\sqrt{\text{Body size}}$									1	15	0.01	n.s.
		$1/\sqrt{\%RNA}$									1	11	0.94	n.s.
		$1/\sqrt{\%DNA}$									1	6	0.01	n.s.
		$1/\sqrt{RNA:DNA}$									1	6	0.10	n.s.
		$1/\sqrt{\%P-TNAs}$									1	6	0.68	n.s.

Significant results (*p*-value < 0.05) are indicated in bold; n.s., not significant.
